# Characterization of a putative metal-dependent PTP-like phosphatase from *Lactobacillus helveticus* 2126

**DOI:** 10.1007/s10123-023-00390-w

**Published:** 2023-06-26

**Authors:** Paul Priyodip, Seetharaman Balaji

**Affiliations:** Department of Biotechnology, Manipal Institute of Technology, Manipal Academy of Higher Education, -576104 Manipal, Karnataka India

**Keywords:** Phytate, *Lactobacillus helveticus*, Protein tyrosine phosphatase, Homology modeling, Phytase, Probiotic

## Abstract

To date, there are very limited reports on sequence analysis and structure-based molecular modeling of phosphatases produced by probiotic bacteria. Therefore, a novel protein tyrosine-like phosphatase was characterized from *L. helveticus* 2126 in this study. The purified bacterial phosphatase was subjected to mass spectrometric analysis, and the identity of constructed sequence was analyzed using peptide mass fingerprint. The 3-D structure of protein was elucidated using homology modeling, while its stability was assessed using Ramachandran plot, VERIFY 3D, and PROCHECK. The bacterium produced an extracellular phosphatase of zone diameter 15 ± 0.8 mm on screening medium within 24 h of incubation. This bacterial phosphatase was highly specific towards sodium phytate as it yielded the lowest K_m_ value of 299.50 ± 4.95 μM compared to other phosphorylated substrates. The activity was effectively stimulated in the presence of zinc, magnesium, and manganese ions thereby showing its PTP-like behavior. The phosphatase showed a molecular mass of 43 kDa, and the corresponding M/Z ratio data yielded 46% query coverage to *Bacillus subtilis* (3QY7). This showed a 61.1% sequence similarity to *Ligilactobacillus ruminis* (WP_046923835.1). The final sequence construct based on these bacteria showed a conserved motif “HCHILPGIDD” in their active site. In addition, homology modeling showed a distorted Tim barrel structure with a trinuclear metal center. The final model after energy minimization showed 90.9% of the residues in the favorable region of Ramachandran’s plot. This structural information can be used in genetic engineering for improving the overall stability and catalytic efficiency of probiotic bacterial phosphatases.

**Supplementary Information:**

The online version contains supplementary material available at 10.1007/s10123-023-00390-w.

## Introduction

Phytic acid (also known as myo-inositol hexakisphosphate) is the principal storage form of phosphorus in plant-based foods such as cereals and legumes (Cosgrove 1966). It mostly exists in salt forms collectively referred to as “phytate.” It is a highly electronegative chemical structure with twelve replaceable protons (Tsao et al. 1997). Therefore, it acts as an antinutrient chelating divalent metallic ions such as zinc, iron, magnesium, copper, manganese, calcium (Nissar et al. 2017), carbohydrates (Thompson et al. 1987), lipids (Mora-Boza et al. 2019), and proteins (Cheryan and Rackis 1980). Phytases (myo-inositol hexakisphosphate phosphohydrolases) catalyze the hydrolysis of phytate into inositol and free orthophosphates (Wyss et al. 1999). However, monogastric animals such as humans, swine, and poultry lack sufficient phytase-producing bacteria in their intestines (Balaban et al. 2014). This phytate-linked mineral deficiency leads to several nutritional deficiency disorders.

Based on the structure, phytases are classified into four classes: They are histidine acid phosphatases (HAPs), beta-propeller phytases (BPPs), purple acid phosphatase (PAPs), and cysteine protein tyrosine phosphatases (PTPs) (Mullaney and Ullah 2003). The HAPs are mostly characterized from bacteria, fungi, and plants, and share two common conserved motifs “RHGXRXP” and “HD” near N and C termini, respectively. The mode of catalysis involves a two-step mechanism where the guanidino group of an arginine residue in the ‘RHG’ motif activates the incoming phosphate of phytate. The stimulated electrophile is attacked by histidine leading to the product formation (Van Etten et al. 1991; Ullah et al. 1991; Oh et al. 2004). The presence of EDTA stimulates HAPs at optimum pH in the range between 4.0 and 4.5 (Gontia-Mishra and Tiwari 2013). Some of the HAPs have been reported for *Aspergillus niger* (Weaver et al. 2009), *Sporotrichum thermophile* (Singh and Satyanarayana 2009), *Arabidopsis thaliana* (Mullaney and Ullah 1998), and *Escherichia coli* (Greiner et al. 1993). The BPPs are the second class of phytases mostly of *Bacilli* genera. They have two phosphate-binding (catalytic and affinity) sites and six calcium-binding sites. The binding of two phosphate groups activates the enzyme; it is followed by the binding of three calcium ions into the catalytic and affinity sites, respectively. Catalysis is initiated by the attack of hydroxyl ion of a water molecule on the first phosphate. There, every alternate phosphate group of phytate is cleaved (Ha et al. 2000; Shin et al. 2001). The BPPs show optimal activity in the alkaline pH region from 7.0 to 8.0 and are well characterized in *Bacillus amyloliquefaciens* (Oh et al. 2001). The purple acid phosphatases (PAP) have five conserved regions, DxG/GDx2/GNH(E, D)/Vx2H/GHxH (Schenk et al. 2000). The PAPs of animal origin have two ferric ions while those from plants have one iron and a zinc/magnesium in their active site. The N-terminal of the active site is made up of antiparallel beta-sheets, while the C-terminal contains both alpha helices and beta sheets. The presence of aromatic amino acid tyrosine gives purple color to these enzymes (Gontia-Mishra and Tiwari 2013; Antanaitis and Aisen 1983). They show optimal activity in the acidic pH region in between 4.0 and 5.5 and are of molecular masses 35 to 55 kDa (Lei et al. 2007). The PAPs are characterized from soybean (Hegeman and Grabau 2001) and *Aspergillus niger* (Mullaney et al. 1995). The fourth class of phytases is cysteine tyrosine phosphatases also known as PTPs. They are further categorized into receptor-like PTPs, intracellular PTPs, and dual-specificity PTPs. Receptor-like PTPs have an extracellular membranous domain in addition to one or two catalytic domains. The intracellular PTPs have their catalytic domain in the amino-terminal (Stone and Dixon 1994), while the active site of dual PTPs has cysteine-containing motif HCxxGxxR(T/S) forming a P loop (Lei et al. 2007). This loop acts as the substrate-binding pocket whose depth determines the level of phytate specificity. A conserved arginine residue folds in the above pocket to help in substrate attachment and catalysis (Denu and Dixon 1998). Catalysis is initiated by nucleophilic cysteine on the phosphate group of the phytate molecule resulting in a thiophosphate enzyme intermediate (Chu et al. 2004). This molecule is hydrolyzed by water releasing phosphate resulting in myo-inositol monophosphate in the process (Walton and Dixon 1993). They show optimal activity in the acidic pH between 4.5 and 6.5 and need lead ions for stimulation (Lei et al. 2007).

These PTPs are part of a bigger DNA polymerase group whose N-terminal conformation resulted in a polymerase and histidinol family of phosphoesterases (PHP) superfamily. Therefore, PHP is a subgroup of PTPs and is mostly present in bacteria as seen in the case of *Streptococcus pneumoniae* (Aravind and Koonin 1998). The active site of these enzymes contains four conserved motifs: “18-GxFKY-22,” “45-RG-46,” “75-GGGx[RK]IxH-81,” and “92-GxSxxxGxAxH-102,” respectively (Klumpp et al. 2002). The presence of arginine (Arg 139) helps in the attachment of the substrate to the enzymatic active site such that the phosphorus group is above the nucleophile. This reaction removes all the existing negative charge from the phosphorus atom making it susceptible to nucleophilic attack. The hydroxyl ion generated from metal-bound water molecules attacks the above phosphorus (Hagelueken et al. 2009). The phytate-PHP complex is stabilized by the amine group of lysine (Lys-21) during the transition state. This is followed by phosphate liberation from phytate (Gong et al. 2009). However, till date, there are very limited reports on PTPs of Lactobacillli. Therefore, the present study is focused on the characterization of a putative PTP from *Lactobacillus helveticus* 2126.

## Materials and methods

### Bacterial culture and maintenance

Bacterium *Lactobacillus helveticus* 2126 was procured from the National Collection of Industrial Microorganisms (NCIM), National Chemical Laboratory (NCL), Pune, India. The strain was revived in deMan Rogosa Sharpe (MRS) medium at 37 °C under constant agitation at 180 rpm. The culture was preserved in the form of agar plates and slant cultures.

### Qualitative phytase identification

The bacterium was grown in a phosphatase screening medium (PSM) (Priyodip and Balaji 2018). In this study, phytate was used as the sole carbon source in place of dextrose. Moreover, phytate can serve as a phosphate source. Bacterial culture (100 μL) was inoculated in PSM agar plates followed by incubation at 37 °C for 24 h. The visualization of a zone confirms extracellular bacterial phytase activity. The average mean zone diameter as a result of three independent experiments was used for interpretation.

### Protein estimation

The bacterial culture (~1 mL) was inoculated in 100 mL of PSM broth and incubated at 37 °C under constant shaking at 170 rpm for 24 h. It was subjected to centrifugation at 10,000 rpm for 10 min at 4 °C. The supernatant containing total protein is subjected to Bradford assay for finding its concentration.

### Phosphatase assay

The assay was conducted by ammonium molybdate method. Equivalent volumes of 6.25 mM sodium phytate and bacterial supernatant were mixed and incubated at 37 °C for 30 min. After reaction inhibition with 5% trichloroacetic acid (TCA), 1 mL of color reagent (ammonium phosphomolybdic acid) was added to the reaction mixture. The concentration of blue-colored complex was evaluated at 700 nm. The substrate specificity of bacterial phosphatase was measured in the presence of six phosphorylated substrates.

### Effect of modulators

The effect of modulators on enzymatic activity were studied at different concentrations by pre-incubating the enzyme with these for 15 min before the assay (Suleimanova et al. 2015). The experiments were conducted in triplicates and mean absorbance readings were used for calculations. Relative enzymatic activities were expressed in terms of percentage, and assay results in the absence of modulators were considered as control (100%).

### Enzyme purification

The supernatant containing extracellular phytases was subjected to 90% ammonium sulfate precipitation followed by ultrafiltration. The partially purified enzyme was passed through a gel filtration column, and the resulting fractions were separated using SDS PAGE.

### Trypsin digestion

This procedure was carried out at the Molecular Biophysics Unit, Indian Institute of Science, Bangalore, India, and followed a similar procedure as reported in Granvogl et al. (2007) and Lung et al. (2008). For this, the purified protein gel band was excised and transferred into a microcentrifuge tube containing sterilized distilled water to keep it hydrated. The gel piece was washed with 500 μL of wash solution containing 50% acetonitrile and 50 mM ammonium bicarbonate. This sample was incubated at room temperature for 15 min under slow agitation. The Coomassie dye solution was completely removed by repeated washing (twice for 15 min). The gel was dehydrated in 100% acetonitrile for 5 min to give an opaque white color. The excess acetonitrile was removed by drying in an evaporator for 15–20 min. Then, the sample was placed in a 150-μL reduction solution made up of 10 mM dithiothreitol (DTT) and 100 mM ammonium bicarbonate for 30 min at 56 °C. The reduction solution was replaced by an influx of 100 μL of alkylation solution (50 mM iodoacetamide, 100 mM ammonium bicarbonate). The sample was incubated at 37 °C under dark for 30 min. The alkylation solution was discarded, and the gel was washed with 500 μL wash solution for 15 min under mild agitation at room temperature. The gel after wash was again dehydrated in 100 μL of 100 μL of 100% acetonitrile for 5 min. The acetonitrile solution was discarded, and the gel was dried in an evaporator. Protease digest solution (20 μg of lyophilized trypsin dissolved in 1 mL of 50 mM ammonium bicarbonate stored at −70 °C) in 20 μL quantity was used to rehydrate the gel overnight at 37 °C. The sample was subjected to centrifugation at 12,000 g for 30 s. The supernatant containing fragmented tryptic peptides was transferred to a centrifuge tube, where 25–50 μL of extraction solution (60% acetonitrile, 0.1% trifluoroacetic acid, TFA) was added. The samples were then subjected to ultrasonication for 10 min. The samples were then again centrifuged at 12,000 g for 30 s. The samples were again treated with extraction solution, followed by ultrasonication for 10 min and supernatant preservation.

### Mass spectrometric analysis

The procedure was carried out similar to the one reported in Kerovuo et al. (1998) and Lung et al. (2008). The fragmented peptides were dried in an evaporator, and 5 μL of resuspension solution (50% acetonitrile, 0.1% trifluoroacetic acid, TFA) was added to it. This was subjected to ultrasonication and gently agitated. The sample was centrifuged, and 0.5 μL was introduced in the matrix-assisted laser desorption/ionization (MALDI) plate having 0.5 μL of an alpha-cyano-4-hydroxycinnamic acid matrix (10 mg/mL in 50% acetonitrile, 0.1% TFA). The spots were allowed to dry and then loaded into the voyager UltrafleXtreme MALDI time-of-flight (TOF)/TOF (Bruker Daltonics, Germany) fitted with a flash detector. The process was calibrated using internal tryptic peaks of 842.5 and 2211.1 Da in the mass range from 600 to 3800 Da. Raw data obtained in reflectron mode was analyzed using Flex Analysis software provided by the manufacturer reported as monoisotopic masses. The search engine Mascot sequence query (version 2.5; Matrix Science, London, UK) was used to process mass spectrometric (MS) data. The first search was performed under *Bacillus subtilis* category with default parameters: peptide tolerance of 1.2 da, 1 missed cleavage, NCBIprot database, and MH^+^ peptide charge state. De novo sequencing was carried out on amino acid data obtained from Mascot search results using National Center for Biotechnology Information (NCBI) BLAST, FASTA.

### Sequence similarity

The peptide mass fingerprint showed 46% query matches with tyrosine-protein phosphatase belonging to homologous organism *Bacillus subtilis* (WP_014478195). Since there was no sequence entry corresponding to *L. helveticus* 2126, the phosphatase sequence was reconstructed based on its structural homologs *B. subtilis* and *Ligilactobacillus ruminis* (WP_046923835.1). They both share sequence similarity of 73.9% with 257 amino acid overlaps. The sequence similarity search in NCBI BLASTp yielded tyrosine-protein phosphatase of *B. subtilis* as the homologous sequence match from the Protein data bank (PDB).

### Phylogenetic analysis

The evolutionary history was inferred using the Minimum Evolution (ME) Method. The evolutionary distances were computed using the Poisson correction method and are expressed in terms of number of amino acid substitutions per site. The ME tree was searched using the Close-Neighbor-Interchange (CNI) algorithm (Thomas 2001) at a search level of 1. The neighbor-joining algorithm was used to generate the initial tree. This analysis involved 100 amino acid sequences. All ambiguous positions were removed for each sequence pair (pairwise deletion option). There was a total of 282 positions in the final dataset. Evolutionary analyses were conducted in MEGA1111.0.10 (Tamura et al. 2021).

### Homology modeling

DeepView (Swiss-PDB viewer) is used in project mode (semi-automatic method) to build a homology model of the *Lactobacillus helveticus* phosphatase (LHP). The target sequence is wrapped (threaded) onto the 3D template (PDB: 3qy7), and the threading energy was calculated. The sequence alignment was manually edited by introducing gaps (indels) to improve the physicochemical properties of amino acids. The coordinates of template were assigned to the target using online submission made to the Swiss model server. The structure was aligned using the Swiss-PDB viewer and submitted to SWISS-MODEL as a project file.

### Loop generation

The loop regions of the predicted LHP model were fixed by a new loop conformation using the “Build Loop” option of DeepView. The process uses GROMOS Force-Field as well as the mean force potential as an energy function. Besides loop building, there is another option available in the DeepView, i.e., “Scan Loop” to identify similar loops based on the sequence similarity from the PDB loop database. The similarity score for the loop region was calculated using the PAM200 matrix. The best possible loop was manually selected based on the parameters such as incorrect phi/psi angles and clash score. The selected loop was inserted and performed using an energy minimization step. The model was submitted to assess Ramachandran’s plot quality.

### Structure validation

The quality of the proposed LHP model was assessed by using VERIFY 3D and PROCHECK. The VERIFY 3D program assessed the protein model based on a 3D-profile, whereas the PROCHECK assessed various parameters such as the main and side-chain parameters, Chi1-Chi2, and Ramachandran plots by residue type, residue properties, main-chain bond length distributions, main-chain bond angle distributions, RMS distances from planarity, and distorted geometry. All these properties are displayed in the form of plots.

## Results and discussions

### Phytate hydrolysis

**Fig. 1:**
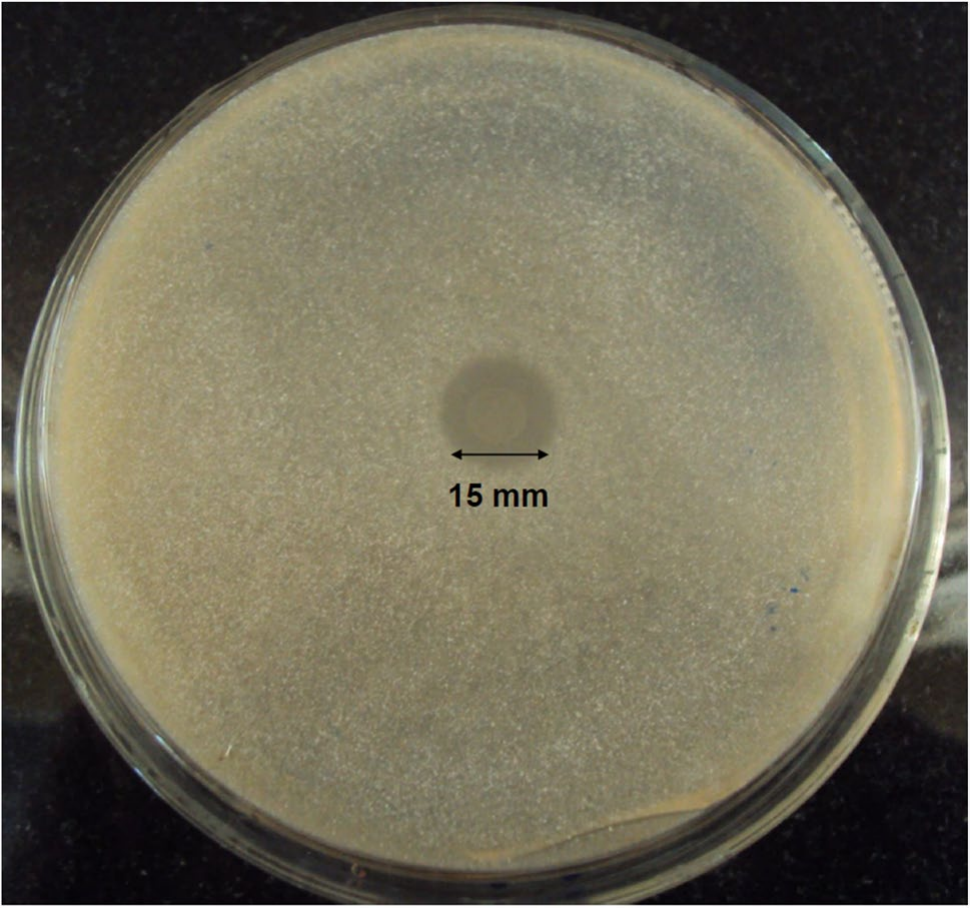
Phosphatase activity of *L. helveticus* 2126 on PSM plate

The bacterium *L. helveticus* 2126 produced an extracellular phosphatase and effectively hydrolyzed phytate with a 15 ± 0.8 mm zone of clearance (Fig. 1).

Phytate induced the production of extracellular phytase within 24 h of incubation in the present study. A similar result was obtained for other bacterial phytases reported in Priyodip and Balaji (2018). However, most of the bacterial phytase activities reported to date were observed after a longer incubation. This was seen in the case of *Bacillus* sp. and *Pseudomonas* sp. P6., where the phytate utilization zones of diameters 11 mm and 32 mm were obtained after 96 and 72 h, respectively (Demirkan et al. 2014; Singh et al. 2013). The bacterium *L. helveticus* 2126 was tested for phytate utilization as the sole carbon and phosphate source for its growth. Phytate was also used as the limiting nutrient for *Burkholderia* sp. (Graminho et al. 2015), *Klebsiella pneumoniae* (Escobin-Mopera et al. 2012), and *Bacillus subtilis* (Kerovuo et al. 1998), showing specific phytase activities of 1.6 U/mg, 5.64 U/mg, and 8 U/mg, respectively.

### Substrate specificity studies

The phosphatase from *L. helveticus* 2126 (LHP) showed highest substrate specificity towards sodium phytate as compared to other phosphorylated compounds (Table S1) (see supplementary material). This was proved by the lowest Michaelis-Menten constant (K_m_) value of 299.50 ± 4.95 μM in comparison with 320.54 ± 12.03 μM obtained for sodium hexametaphosphate, 2011.89 ± 123.85 μM for phenyl phosphate, 875.19 ± 82.77 μM for α-D-glucose-6 phosphate, 343.02±10.06 μM for Inosine 5′ monophosphate, and 1124.39 ± 77.01 μM for pyridoxal 5′ phosphate. Similar phytate-specific bacterial phytases were reported for *Bacillus subtilis* with K_m_ 293 μM (Jain et al. 2018), *Sporosarcina globispora* with K_m_ 254.90 ± 8.52 μM (Priyodip and Balaji 2018), *Bacillus amyloliquefaciens* with K_m_ 550 μM (Kim et al. 1998), *Bacillus* spp. with K_m_ 50 μM (Choi et al. 2001), and *Escherichia coli* with K_m_ 130 μM (Greiner et al. 1993), respectively. To date, there are limited reports on phytate-specific phytases from Lactobacilli. Broad-specific phosphatases were characterized from *Lactobacillus* spp., *L. sanfranciscensis*, and *L. plantarum* with higher specific activities of 120.2 U/mg and 0.815 U/mg for *p*-nitrophenyl phosphate as compared to 96.8 U/mg and 0.463 U/mg obtained for sodium phytate (Graminho et al. 2015; Escobin-Mopera et al. 2012).

### Effect of modulators

The presence of natural chemicals such as citrate, tartrate, and metallic ions iron (Fe^3+^), zinc, calcium, copper, sodium, and potassium had a variable effect on bacterial phytase activity (Table S2). At lower concentrations, citrate (0.05 M) and tartrate (0.10 M) did not influence phytase activity. However, at a higher concentration of 0.50 M, enzymatic catalysis was strongly inhibited by 35.02% and 37.12%, respectively. The presence of citrate slightly enhanced bacterial phytase activity by 2.60%, while tartrate inhibited the same by 0.80% at 5 mmol/L (Lan et al. 2011). In a similar study on bacterial phytase, citrate and tartrate failed to affect catalytic activity in *Enterobacter* sp. (Greiner and Farouk 2007). Phytase activity in *L. helveticus* 2126 was slightly stimulated by ferric ions at 0.05 M (1.08%) and 0.1 M (3.24%) but was inhibited by the same at 0.5 M (6.82%). Phytase inhibition by 29.2% at 0.10 mM and 32.40% at 1.0 mM was also observed in the case of *Bacillus* spp. (Sajidan et al. 2015). Similarly, phytase activity for *Selenomonas ruminantium* was inhibited by 6.50% in the presence of ferric ions at 5 mmol/L (Yanke et al. 1999). The presence of magnesium ions stimulated bacterial phytase activity in *L. helveticus* 2126 by 6.04% at 0.05 M, 12.13% at 0.1 M, and 32.41% at 0.5 M, respectively. These ions strongly inhibited *Lactobacillus plantarum* phytase activity by 28% at 1 mM and 38% at 5 mM (Sumengen et al. 2013). However, magnesium ions at 0.10 and 1.0 mM failed to affect phytase activity in *Bacillus* spp. (Sajidan et al. 2015). Calcium ions in lower concentrations stimulated *L. helveticus* phytase activity by 2.08% at 0.05 M and 8.87% at 0.1 M. However, at a higher concentration of 0.5 M, the activity was reduced by 5.63%. A similar result was observed for *Bacillus* spp. (Sajidan et al. 2015), where calcium ions at 0.1 M and 1 mM enhanced phytase activity by 14.7% and 15.3%, respectively. Phytase activity was, however, strongly inhibited by 17.1% and 38% in the presence of calcium ions at 0.5 and 1 mM (Sarıbuga et al. 2014). Also, for *Lactobacillus brevis*, activities were inhibited by 25% at 1 mM and 35% at 5 mM, respectively (Sümengen et al. 2012). Copper ions at a lower concentration of 0.05 M did not influence bacterial phytase activity. However, at 0.1 and 0.5 M, the activities were inhibited by 27.93% and 43.02%, respectively. Similar inhibitory activity of 23% was observed for *Lactobacillus sanfranciscensis* in the presence of copper ions (De Angelis et al. 2003). Copper ions at 1 and 5 mM also inhibited phytase activities by 29% and 27% in *L. brevis* (Sümengen et al. 2012). The presence of manganese ion strongly stimulated phytase activity in *L. helveticus* 2126 by 10% at 0.05 M, 21.05% at 0.1 M, and 41.24% at 0.5 M. Similar phytase activation by 19.5% was observed for *Mitsuokella jalaludinii* in the presence of 0.005 M (Lan et al. 2011). At the same metal concentration, phytase activity was stimulated by 2.6% for *Selenomonas ruminantium* (Yanke et al. 1999). In contrast to the above reports, manganese ions at 10^−4^–10^−3^ M did not affect phytase activity of *Enterobacter* sp. (Greiner and Farouk 2007). These ions however stimulated phytase activity by 24% at 1 mM and 35% at 5 mM in *L. brevis* and by 28% and 24% at 1 and 5 mM in *L. plantarum*. Zinc ions strongly enhanced phytase activity in *L. helveticus* 2126 by 9.14% at 0.05 M, 18.25% at 0.1 M, and 40.74% at 0.5 M, respectively. Phytase activity in *L. brevis* was enhanced by 21% by zinc at 5 mM (Sümengen et al. 2012). Sodium ion–stimulated phytase activity in *L. helveticus* 2126 was stimulated by 4.09% at 0.5 M, while potassium ions did not affect the bacterial phytase. Sodium ions also stimulated phytase activity by 4.09% at 0.5 M for *L. brevis* (Sümengen et al. 2012).

### Phytase purification

**Fig. 2:**
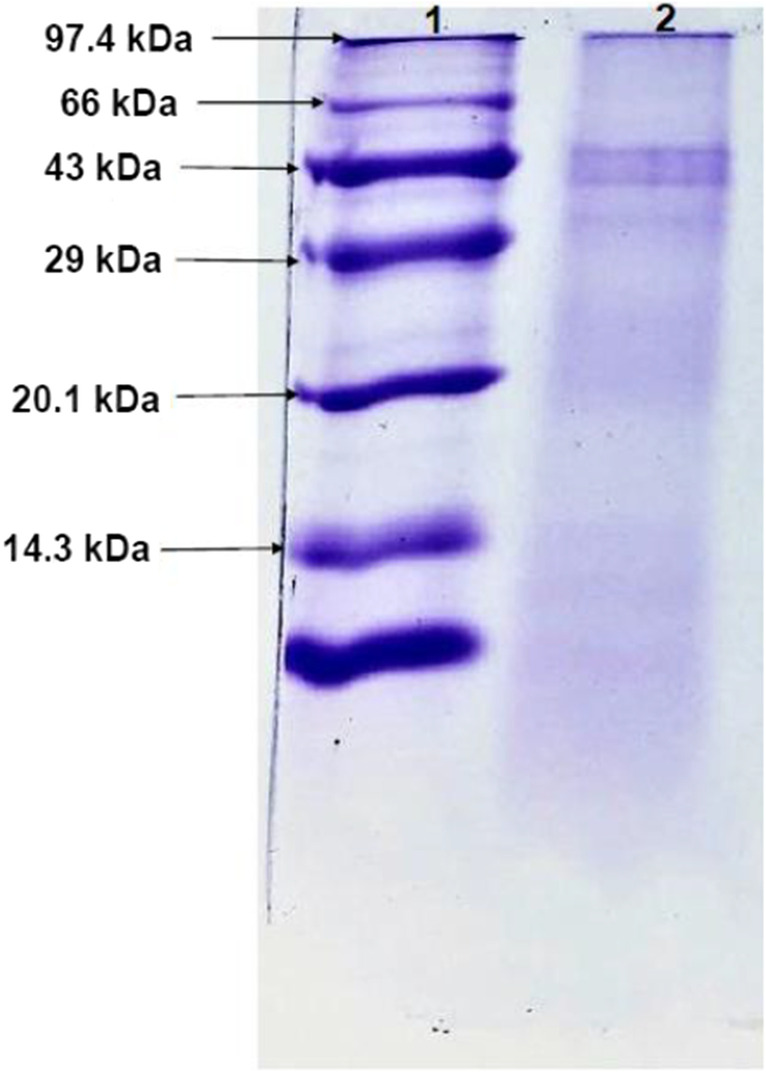
SDS-PAGE; lane 1, Molecular weight marker; lane 2, *L. helveticus* 2126 phosphatase

The bacterium *L. helveticus* 2126 produced a monomeric phytase of molecular mass 43 kDa (Fig. 2).

Similar monomeric enzymes were characterized from *Lactobacillus sanfranciscensis* (De Angelis et al. 2003), *Lactobacillus plantarum* (Sumengen et al. 2013), *Enterobacter* sp., *Geobacillus* sp. (Dokuzparmak et al. 2017), and *Bacillus subtilis* (Jain et al. 2018) with molecular masses of 50, 46, 42, 106.04, and 46 kDa, respectively. However, trimeric intracellular phytases with subunits of 72, 36, and 33 kDa were reported for *Lactobacillus plantarum* (Sumengen et al. 2013). Also, intracellular dimeric phytases were observed for *Lactobacillus brevis* with molecular masses of 73 and 34 kDa, respectively.

### Peptide sequencing

The *m*/*z* data from MS analysis is given in supplementary information (Fig. S1; Table S3). The *m*/*z* data was used as an input for the MASCOT search. The search parameters set are as follows: NCBIprot as the default database. *Bacillus subtilis* category showed a partial match to a tyrosine-protein phosphatase (46% query coverage) from *Bacillus subtilis* with accession number WP_014478195. The corresponding score was 35 with a monoisotopic mass of 28847 and a calculated pI of 6.60. The peptide matches were based on amino acid positions: 28-35, R. AAVRQGIR.T; 32-49, R. QGIRTIIATPHHNNGVYK. N; 69-93, K. EDIPLHVLPGQEIRIYGEVEQDLAK. Q; 83-93, R. IYGEVEQDLAK. Q; 117-139, R. YAEQLFYDLQLKGYIPVIAHPER. N; 142-156, R. EIRENPSLLYHLVEK. G; 202-233, R. NFHTQEALYVLEKEFGSELPYMLTENAELLLR. N.

The mass spectrometric data (*m*/*z* ratio) for *L. helveticus* 2126 phosphatase as mentioned above showed a partial sequence match to *Bacillus subtilis* (PDB id: 3QY7). Moreover, the alignment between PTP from *B. subtilis* and *Ligilactobacillus ruminis* (WP_046923835.1) belonging to a PHP superfamily showed a sequence similarity of 61.1% with 257 amino acid overlaps. Therefore, the *m*/*z* data obtained from the MS spectra helped us to map the matching regions from both *B. subtilis* as well *L. ruminis* to obtain the final sequence construct.

MRFDKIVDLHCHILPGIDDGSKNLETSLELAE**AAVRQGIRTILATPHHNNGVYK**NHKDDVIKVCDDFQTELDN**EDIPLHVLPGQEIRIYGEVEQDLAK**LGIDEQKRYMLLEFPHGDVPG**YAEQLFYDLQLKGYIPVIAHPER**NR**EIRENPSLLYHLVEK**FISNGALAQVTATSYVGGFGEHVAQISHDMVEHNLVQVVASDAHSL**NFHTQEALYVLEKEFGSELPYMLTENAELLLR**LINGDYVAARDYSPIKKKKRFLFF

(*Lactobacillus ruminis —* plain text, and the **bold** text *B. subtilis*)

**Fig. 3:**
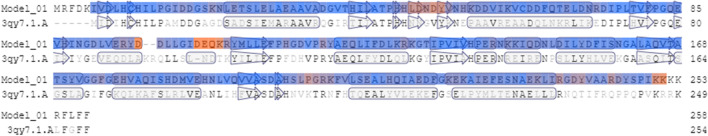
Alignment between sequence construct and template (*B. subtilis*-3QY7) for homology modeling

The sequence construct, when subjected to a protein BLAST search, resulted in sequence identities with 100% query coverage to *Ligilactobacillus ruminis* (75.09% identity), *Lactobacillus ruminis* (72.45% identity), and *Bacillus subtilis* (64.50% identity). When the search was restricted to “PDB database,” the query showed 64.5% identity to *B. subtilis* (id: 3QY6_A) and 29.18% identity to *Streptococcus pneumoniae* (id: 2WJD_A). The PTPs belonging to *B. subtilis* and *S. pneumoniae* are well documented in the literature (Yanke et al. 1999; Hagelueken et al. 2009) and hence were used as templates for modeling LHP. The construct along with its template structure was aligned (Fig. 3).

### ﻿Phylogenetic analysis

**Fig. 4:**
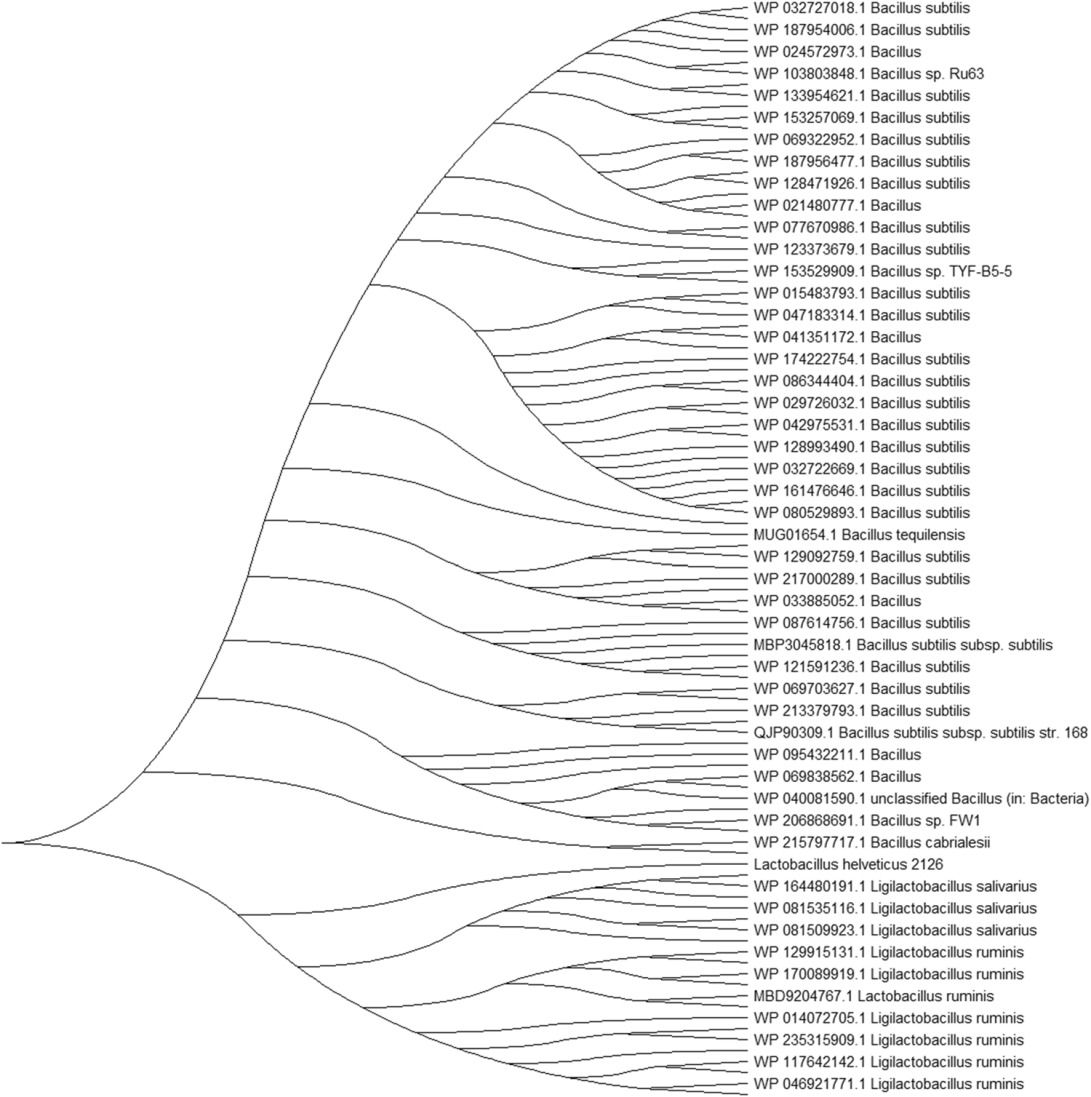
Family tree of LHP

The optimal tree is shown in Fig. 4. This shows the related phosphatase sequences of *L. helveticus* 2126 ranging from *B. subtilis* (WP 032727018.1) to *Ligilactobacillus ruminis* (WP 046921771.1). In the overall arrangement, there are approximately five different clusters mostly comprising of sequences belonging to *B. subtilis*, *Lactobacillus ruminis*, and *Ligilactobacillus ruminis*. The bacterial phosphatase from *L. helveticus* 2126 share phylogenetic relationship with *L. salivarius* spp. (WP_164480191.1, WP_081535116.1, WP_081509923.1) and *Ligilactobacillus ruminis* (WP_129915131.1, WP_170089919.1).

### Protein homology modeling

The sequence-structure alignment: The initial five amino acid residues at the N-terminus are not aligned. In the structurally conserved regions, the alpha-helices and beta sheets are represented by cylinders and arrowheads, respectively. The rest of the sequences are variable regions. The matching residues are shown in bold letters throughout the sequence.

**Fig. 5:**
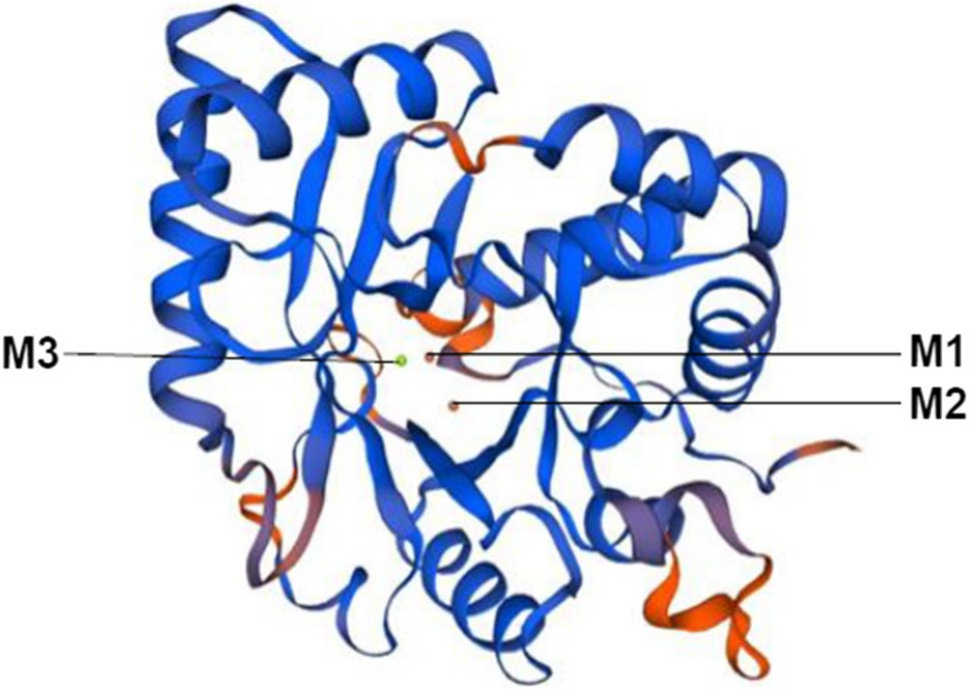
Homology modeled distorted Tim barrel structure of *L. helveticus* 2126 phosphatase with trinuclear metal center

The LHP sequence construct was modeled and validated. The final model showed a distorted TIM-barrel structure with 9 alpha-helices and 7 beta-strands (Fig. 5). A trinuclear metal center retaining the conserved M1, M2, and M3 sites for copper, iron, and manganese ions is shown in the center (Mijakovic et al. 2005).

**Fig. 6:**
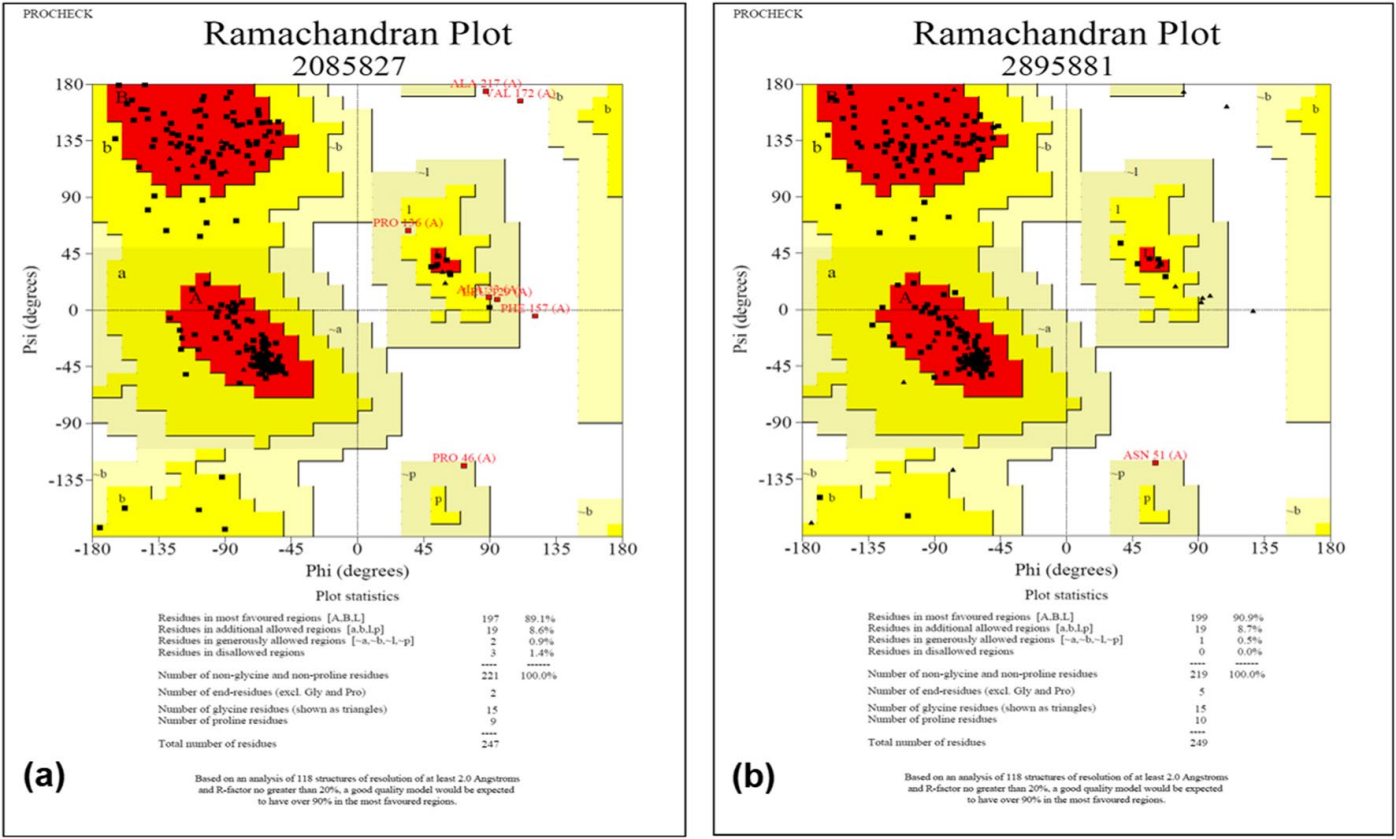
Ramachandran Plot. **a** Protein model before refinement. **b** Protein model after refinement

The most favorable regions (the core regions) of Ramachandran’s plot were 89.1%. Therefore, the structure was iteratively energy minimized to improve the model quality. However, there was no improvement in the core regions of Ramachandran’s plot. The loop regions were identified using Verify 3D for further validation. The conformation of these regions was improved by reducing the steric clash and this was done by using the “build loop” option in the DeepView v4.1.0. Followed by an energy minimization step, the model was submitted to assess Ramachandran’s plot quality. The improved model encompasses 90.9% of the residues in the core regions of Ramachandran’s plot (Fig. 6).

Based on their catalytic mechanism, PTPs are divided into lower molecular weight metal independent PTPs (LMPTPs) and metal-dependent PHP-like PTPs (Denu and Dixon 1998). Their characteristics are dependent on conserved residues in the active, substrate binding, catalytic, and metal-binding sites. However, all share a common active site sequence motif “HCXXGXXR (S/T).” For LHP in the present case, a similar conserved sequence in the form of “HCHILPGIDD” was present at the 10th position. But in the case of *B. subtilis*, the “HCH” headlining motif was observed in the 5th position with a corresponding full sequence “HCHILPAMDD” (Hagelueken et al. 2009). There is a slight shift in the conserved pattern due to genus-specific variations. The arrangement of amino acids may also influence the conformational states of PTP and its catalytic mechanism.

In the LHP, Val-215 is present instead of the catalytic amino acid (Cys-215) as in PTPs. Similarly, His-181 and Asp-188 are present instead of Asp-181 and Asp-188 (as in PTPs), which facilitates the protonation of a tyrosine-containing enzyme. The Asp-188 is in the WPD loop of the active site. It can also be assumed that the amines and guanidium side chain of conserved Arg residue helps in positioning phosphate in the catalytic site thereby stabilizing the negative charge of phytate. In all PTPs, a p-tyr loop containing Tyr-45 is conserved, whereas, in LHP, it was observed in the 53rd position. Their catalytic site contains loop I which has a conserved sequence “Asn44-Glu51”. The conserved motifs (in catalytic, substrate, and metal-binding sites) observed in LHP are due to homology with PTPs of *B. subtilis*, *S. pneumoniae*, and *L. ruminis*.

The bacterial phytase in the present study also has a similar amino acid arrangement in the form of “Asn49-His56” with a slight shift in the base position. Loop II in *B. subtilis* phosphatase has “Ala168-Lys173” and “Lys173,” or similar amino was replaced by “His178” in case of LHP. Both the loops exist in both “open” and “closed” states. In closed conformation, loop I creates a hydrophobic pocket for binding tyrosine (attached substrate). The active site of loop I has a “(G/P)X(Y/F)” motif with a Tyr-53 residue, and it may be needed for the attachment of phytate. In loop II, the active site of PTPs has a conserved motif (G/P)X_1~2_FGX_0~1_(K/R) with a Phe171 residue, whereas, in LHP, Phe178 was present. This may act as a “gatekeeper” modulating the entry of tyrosine-based phytate in the enzymatic active site. The presence of conserved glycine/proline on either side of the Phe178 may aid in overall structural reorganization (Kim et al. 2011). The crystal structures of YwqE suggest the presence of ferric and magnesium ions, in the metal-binding site located at the C-terminal of enzyme (Kim et al. 2011). This is a trinuclear center having M1, M2, and M3 sites for binding metal ions. In Mijakovic et al. (2005), it was suggested that the presence of copper, as well as manganese ions, enhances the phosphatase activity of *Bacillus subtilis*. Thus, these binding sites can be occupied by either of the metal ions. They are surrounded by His (10, 12, 47, 139, and 203 positions), Glu (85, 104, 111, and 233 positions), and Asp (8, 201, and 232 positions).

As previously mentioned, Tyr46 (in YwqE) might occupy Tyr53 (in LHP). However, it is also possible that conserved Tyr48 might have shifted to Tyr53 (in LHP). Also, mutation of Arg139 in *S. pneumoniae*–based PTP (Hagelueken et al. 2009) showed a considerable decline in phytate hydrolysis. Therefore, it might play a key role in catalysis interacting with the incoming water molecule. LHP sequence showed considerable similarity to *L. ruminis* based PTP. However, their crystal structure is still unknown. A recent PTP-like phosphatase reported from *Lactobacillus fermentum* (Sharma et al. 2018) also lacked structural characteristics. Low molecular weight protein tyrosine phosphatases from Gram-negative *Selenomonas ruminantium* (Puhl et al. 2007) describe a similar phosphatase which has significant structural differences as compared to PHP-like PTPs; hence, it was not used for comparison.

## Conclusions

The bacterium *L. helveticus* 2126 effectively utilized phytate as the sole carbon and phosphate source, and produced an extracellular phosphatase. The bacterium was highly specific towards sodium phytate and showed less activity towards other phosphorylated substrates. The bacterial phosphatase was strongly stimulated by zinc, magnesium, and manganese ions, therefore falling into the category of metal-dependent PTP like phosphatases. The phosphatase of ﻿*Lactobacillus helveticus* showed a truncated TIM-barrel structure with a trinuclear metal center. In addition to this, the active region comprising conserved amino acids “HCHILPGIDD” contribute to catalytic and substrate binding sites. This information can be potentially used for overexpression of phytase for treating mineral deficiencies in monogastric animals. This is the first report of a PTP-like phosphatase from *L. helveticus*, and hence, further research is warranted. 

### Supplementary information

ESM 1
