# Supplementary material for: Characterization of a putative metal-dependent PTP-like phosphatase from Lactobacillus helveticus 2126
Source: Int Microbiol. 2023 Jun 26;27(1):37–47. doi: 10.1007/s10123-023-00390-w (PMC10830716; doi:10.1007/s10123-023-00390-w)
Supplement: Supplementary file 1 — ESM 1 [file 10123_2023_390_MOESM1_ESM.doc]

**Table S1**

| **Substrates** | **Kinetic parameters** | | | |
| --- | --- | --- | --- | --- |
|  | **K_m_ (μM)** | **V_max_**  **(μM/min)** | **K_cat_ (min^-1^)** | **K_cat_/K_m_×10^-3^ (min^-1^ µM^-1^)** |
| Sodium phytate | 299.50±4.95 | 9.56±4.10 | 2.055 | 6.86 |
| Sodium  hexametaphosphate | 320.54±12.03 | 9.42±2.05 | 2.025 | 6.31 |
| Phenyl phosphate | 2011.89±123.85 | 14.92±6.13 | 3.2 | 1.59 |
| α-D-glucose-6  phosphate | 875.19±82.77 | 9.17±5.03 | 1.97 | 2.25 |
| Inosine 5'  monophosphate | 343.02±10.06 | 9.39±4.34 | 2.01 | 5.85 |
| Pyridoxal 5'  phosphate | 1124.39±77.01 | 15.17±6.89 | 3.26 | 2.89 |

**Table S2**

| **Bacterium** | **Relative phytase activities (%)** | | | | | | | | | | | | | | | |
| --- | --- | --- | --- | --- | --- | --- | --- | --- | --- | --- | --- | --- | --- | --- | --- | --- |
|  | **citrate** | | | **tartrate** | | | **Fe^3+^** | | | **Mg^2+^** | | | | **Ca^2+^** | | |
|  | **0.05 M** | **0.1 M** | **0.5 M** | **0.05 M** | **0.1 M** | **0.5 M** | **0.05 M** | **0.1 M** | **0.5 M** | **0.05 M** | **0.1 M** | | **0.5 M** | **0.05 M** | **0.1 M** | **0.5 M** |
| ***Lactobacillus helveticus* 2126** | 100±0.02 | 100±0.16 | 64.98±0.56 | 100±0.04 | 100±0.03 | 62.88±0.66 | 101.08±0.16 | 103.24±0.14 | 93.18±0.12 | 106.04±0.18 | | 112.13±0.08 | 132.41±0.14 | 102.08±0.12 | 108.87±0.13 | 94.37±0.11 |

| **Bacterium** | **Relative phytase activities (%)** | | | | | | | | | | | | | | |
| --- | --- | --- | --- | --- | --- | --- | --- | --- | --- | --- | --- | --- | --- | --- | --- |
|  | **Cu^2+^** | | | **Mn^2+^** | | | **Zn^2+^** | | | **Na^+^** | | | **K^+^** | | |
|  | **0.05 M** | **0.05 M** | **0.1 M** | **0.05 M** | **0.1 M** | **0.5 M** | **0.05 M** | **0.1 M** | **0.5 M** | **0.05 M** | **0.1 M** | **0.5 M** | **0.05 M** | **0.1 M** | **0.5 M** |
| ***Lactobacillus helveticus* 2126** | 100±0.13 | 72.07±0.15 | 46.98± 0.12 | 110±0.14 | 121.05±0.18 | 141.24±0.18 | 109.14±0.20 | 118.25±0.10 | 140.74±0.13 | 100±0.12 | 100±0.09 | 104.09±1.32 | 100±0.05 | 100±0.01 | 100±0.04 |

**Figure S1**

**Table S3**

| **m/z** | **S/N** | **Quality Fac.** | **Res.** | **Intens.** | **Area** |
| --- | --- | --- | --- | --- | --- |
| 539.526 | 12 | 18509 | 1170 | 1839 | 1464 |
| 545.546 | 24 | 17271 | 1827 | 3685 | 1742 |
| 606.641 | 10 | 6517 | 1398 | 1489 | 1155 |
| 634.636 | 15 | 4185 | 1568 | 2209 | 1621 |
| 715.333 | 12 | 23862 | 1962 | 1800 | 1175 |
| 832.657 | 28 | 50861 | 1945 | 3923 | 3027 |
| 842.672 | 10 | 15997 | 1627 | 1396 | 1351 |
| 870.668 | 7 | 15106 | 1533 | 977 | 1046 |
| 993.556 | 34 | 44953 | 1979 | 4716 | 4666 |
| 999.617 | 10 | 4133 | 1814 | 1348 | 1394 |
| 1234.682 | 8 | 28881 | 1361 | 1092 | 2502 |
| 1263.866 | 7 | 20927 | 1612 | 936 | 1619 |
| 1277.890 | 9 | 81506 | 1756 | 1274 | 2166 |
| 1302.826 | 8 | 38219 | 1988 | 1125 | 1714 |
| 1373.810 | 7 | 7861 | 2091 | 958 | 1472 |
| 1383.849 | 11 | 15060 | 1592 | 1487 | 3609 |
| 1434.935 | 7 | 25163 | 1528 | 966 | 2589 |
| 1461.030 | 7 | 17110 | 1328 | 828 | 2695 |
| 1476.716 | 8 | 7512 | 812 | 874 | 4191 |
| 1600.018 | 2 | 4481 | 466 | 257 |  |
| 1644.020 | 3 | 3041 | 573 | 365 |  |
| 1659.078 | 5 | 3018 | 1080 | 743 |  |
| 1701.199 | 7 | 8562 | 946 | 783 | 4245 |
| 1708.182 | 13 | 18839 | 2296 | 2061 | 4371 |
| 1741.520 | 6 | 8296 | 1282 | 463 |  |
| 1766.189 | 5 | 2981 | 1028 | 770 |  |
| 1798.493 | 7 | 18068 | 885 | 776 | 4925 |
| 1839.429 | 10 | 25606 | 1414 | 1276 | 5200 |
| 1874.972 | 9 | 8412 | 911 | 939 | 6564 |
| 1892.794 | 7 | 1248 | 3514 | 1066 | 1819 |
| 1942.512 | 19 | 23749 | 1097 | 2324 | 13001 |
| 1966.568 | 4 | 2913 | 781 | 657 |  |
| 1994.867 | 47 | 20099 | 1102 | 4881 | 29977 |
| 2019.507 | 6 | 3824 | 1280 | 1004 | 2019.507 |
| 2031.602 | 5 | 7556 | 996 | 480 |  |
| 2048.613 | 3 | 9841 | 588 | 216 |  |
| 2084.679 | 6 | 7281 | 1160 | 518 |  |
| 2099.730 | 4 | 3141 | 736 | 625 |  |
| 2152.665 | 3 | 7145 | 7145 | 526 |  |
| 2186.759 | 3 | 8025 | 611 | 340 |  |
| 2213.118 | 12 | 7360 | 954 | 1511 | 11349 |
| 2237.085 | 5 | 3267 | 936 | 864 |  |
| 2269.959 | 4 | 6277 | 705 | 519 |  |
| 2287.911 | 4 | 7521 | 706 | 525 |  |
| 2315.055 | 5 | 5159 | 832 | 684 |  |
| 2332.943 | 6 | 6853 | 1115 | 710 |  |
| 2343.997 | 11 | 86434 | 2126 | 1205 | 4729 |
| 2369.731 | 7 | 41062 | 1202 | 627 | 4238 |
| 2383.048 | 7 | 114 | 8984 | 874 | 770 |
| 2384.883 | 31 | 43574 | 2518 | 3745 | 11794 |
| 2399.932 | 6 | 5500 | 1446 | 513 | 3199 |
| 2442.142 | 4 | 3569 | 782 | 668 |  |
| 2511.658 | 12 | 6174 | 1603 | 1243 | 6618 |
| 2567.292 | 4 | 6909 | 689 | 600 |  |
| 2624.190 | 5 | 6638 | 770 | 658 |  |
| 2665.435 | 3 | 4751 | 558 | 425 |  |
| 2706.319 | 28 | 175381 | 2995 | 2662 | 8729 |
| 2718.287 | 34 | 126504 | 2946 | 3262 | 10698 |
| 2762.640 | 6 | 1026 | 5859 | 542 | 1143 |
| 2809.310 | 6 | 14419 | 1094 | 399 | 3931 |
| 2828.452 | 4 | 5830 | 636 | 560 |  |
| 2842.462 | 4 | 5724 | 579 | 460 |  |
| 2860.447 | 2 | 6091 | 262 | 175 |  |
| 2891.589 | 4 | 4736 | 536 | 498 |  |
| 2905.862 | 9 | 2889 | 1598 | 636 | 4288 |
| 2923.533 | 2 | 6525 | 282 | 177 |  |
| 3020.629 | 2 | 12820 | 287 | 122 |  |
| 3055.811 | 7 | 5292 | 834 | 715 |  |
| 3069.570 | 5 | 6261 | 594 | 426 |  |
| 3173.853 | 2 | 5671 | 204 | 144 |  |
| 3225.615 | 2 | 5738 | 204 | 191 |  |
| 3252.993 | 2 | 6243 | 242 | 202 |  |
| 3267.852 | 19 | 17999 | 1679 | 903 | 7109 |
| 3312.019 | 6 | 1200 | 6571 | 314 | 706 |
| 3334.787 | 4 | 7286 | 375 | 295 |  |
| 3350.119 | 1 | 23244 | 136 | 42.7 |  |
| 3367.730 | 2 | 7240 | 144 | 91.5 |  |
| 3396.757 | 3 | 12255 | 301 | 162 |  |
| 3528.745 | 2 | 8428 | 138 | 89.5 |  |
| 3556.778 | 2 | 4941 | 170 | 157 |  |
| 3797.980 | 7 | 3941 | 447 | 667 |  |
| 3813.106 | 6 | 3975 | 366 | 612 |  |
| 3854.948 | 3 | 5931 | 147 | 125 |  |
| 4094.921 | 3 | 4607 | 137 | 138 |  |
| 4862.544 | 3 | 5908 | 106 | 95.8 |  |
| 4973.792 | 6 | 3923 | 173 | 264 |  |
